# ADHD-related neurodiversity and innovation in marketing teams: a grounded theory framework of customer-oriented boundary-spanning

**DOI:** 10.3389/fpsyg.2026.1777210

**Published:** 2026-10-02

**Authors:** Sima Alipour, Abbas Ali Rastgar, Shahryar Sorooshian

**Affiliations:** 1 Faculty of Economics, Management and Administrative Sciences, Semnan University, Semnan, Iran; 2 Department of Business Administration, University of Gothenburg, Gothenburg, Sweden; 3 Faculty of Engineering and Sustainable Development, University of Gävle, Gävle, Sweden

**Keywords:** ADHD, marketing, Mediated Empathy in Boundary-Spanning, Neurodiverse Cognitive Synergy, Resilient Organizational Adaptation

## Abstract

**Introduction:**

This study explores how ADHD-related neurodiversity is experienced within marketing teams, particularly in customer-oriented and boundary-spanning work.

**Method:**

Using a grounded theory approach, semi-structured interviews were conducted with 35 marketing managers, frontline employees, and freelancers/consultants, including 19 participants with a formal ADHD diagnosis and 16 participants with direct professional experience working with colleagues with ADHD.

**Results:**

The analysis generated three theoretical codes: Neurodiverse Cognitive Synergy, Mediated Empathy in Boundary-Spanning, and Resilient Organizational Adaptation. The findings indicate that ADHD-related characteristics, including divergent thinking, creativity, intense focus, and adaptability, may be experienced as resources. At the same time, impulsivity, distraction, stress, communication difficulties, and social isolation may create challenges. These outcomes appear to depend on task demands, role fit, interpersonal relationships, and organizational support.

**Contribution:**

The study develops an exploratory, data-grounded framework that explains how organizational and relational conditions may shape how ADHD-related characteristics are engaged in customer-oriented marketing work, without assuming a direct or causal relationship between ADHD and innovation.

## 1 Introduction

Neurodiversity—an emerging and dynamic concept in organizational and marketing studies—refers to neurocognitive differences associated with neurodevelopmental conditions such as attention-deficit/hyperactivity disorder (ADHD), autism, dyslexia, Tourette syndrome, and other forms of neurodivergence (Fletcher-Watson, 2022; Apperly et al., 2024). Originating from the autism advocacy movement (Blume, 1998; Singer, 1999), the concept redefines neurological differences as an integral component of natural human diversity by challenging traditional perspectives based primarily on pathology and clinical labels (Wiklund et al., 2018; Curseu et al., 2015; Gardenswartz et al., 2010; van Rijswijk et al., 2024a, 2024b). Unlike traditional medical models that emphasize impairment, the neurodiversity paradigm highlights the diverse ways in which neurocognitive characteristics may manifest as strengths or challenges contingent upon context (Krzeminska and Hawse, 2020). In recent decades, organizations have increasingly recognized the importance of workplace diversity and inclusion and have developed diversity management practices intended to create conditions in which neurodivergent employees can participate and contribute effectively (Austin and Pisano, 2017; Volpone et al., 2022). However, existing research offers a more complex picture than a simple strengths-based account suggests, as the organizational consequences of neurodivergence may depend on task requirements, interpersonal relationships, and the degree of organizational support available. Recent research increasingly conceptualizes workplace neurodiversity as an organizational issue, emphasizing the need to move beyond deficit-oriented perspectives and to recognize how neurodivergent characteristics may represent both strengths and challenges depending on organizational contexts and practices (Silver et al., 2023; Lerner, 2024; Hartman and Hartman, 2024; Tomczak et al., 2025).

Research on neurodiversity suggests that cognitive differences can create opportunities for divergent thinking, knowledge exchange, and alternative approaches to problem-solving, while also creating potential interpersonal and coordination challenges (Mathuki and Zhang, 2022; Qi et al., 2022). This issue is especially relevant to marketing teams, whose work entails creative problem-solving and frequent interactions with customers and other external stakeholders (Wagner et al., 2025). The Customer-oriented boundary-spanning framework—which emphasizes employees’ interactions with customers and stakeholders—thus provides a valuable context for examining how neurodivergent characteristics are experienced and interpreted in roles that require both internal collaboration and external interaction (Bettencourt and Brown, 2003; Wagner et al., 2025). Its three dimensions—external representation, internal influence, and service delivery—highlight different forms of interaction through which employees communicate organizational value, exchange ideas, and respond to customer needs (Bettencourt and Brown, 2003). Rather than assuming that neurodiversity necessarily improves these outcomes, this perspective allows examination of the conditions under which neurodivergent characteristics may be experienced as enabling or constraining resources.

A key issue concerns the relationship between ADHD-related characteristics and work processes. Individuals with ADHD may experience characteristics such as impulsivity, hyperactivity, and periods of intense attention to tasks of interest (Yu et al., 2021). Previous research has associated some of these characteristics with entrepreneurial activity, opportunity recognition, risk-taking, and divergent approaches to problem solving (Wiklund et al., 2016; Lerner et al., 2019; Hatak et al., 2021; Nicolaou et al., 2011). However, these associations should not be construed as evidence that ADHD inherently engenders creativity or innovation. The same characteristics may create difficulties when work requires sustained attention, coordination, emotional regulation, or conventional forms of interpersonal interaction. Relational challenges associated with neurodevelopmental differences may therefore constrain participation and collaboration, particularly in marketing environments where social interaction and team coordination are central (Chapman, 2021; Dwyer, 2022). The co-occurrence of potentially enabling and constraining experiences implies that the organizational consequences of ADHD are contingent rather than uniformly positive or negative.

Creating an inclusive organizational environment is therefore important for understanding how neurodiversity is experienced in teams (Holmes et al., 2021; Volpone et al., 2022). Supportive social interactions and appropriate boundary-spanning practices may help employees navigate relational demands and participate more effectively in collaborative activities (Curseu et al., 2015). Boundary-spanning activities involve proactive interactions with internal and external stakeholders to exchange information, access resources, and respond to external demands (Marrone, 2010; Chien et al., 2021; van Rijswijk et al., 2024a, 2024b). Recent research suggests that boundary-spanning teams may also face interpersonal and coordination demands that can be mitigated through appropriate boundary-buffering practices (Rodríguez-Escudero et al., 2025). These activities may provide both opportunities and challenges for neurodivergent employees: they can expose employees to diverse information and perspectives while simultaneously increasing interpersonal and coordination demands. Existing research has seldom examined how these dynamics manifest in marketing teams, particularly from the perspectives of those directly involved in neurodiverse work contexts. This gap is significant because marketing work integrates creative problem-solving with substantial interpersonal and customer-facing interaction.

Open innovation offers a complementary perspective by conceptualizing innovation as entailing knowledge flows across organizational boundaries (Chesbrough, 2003; West and Bogers, 2017). Recent research further highlights the importance of cross-boundary knowledge flows and interactions in shaping contemporary open innovation processes (Bertello et al., 2024). In marketing contexts, interactions with customers, suppliers, partners, and other external actors can provide access to diverse information and ideas. Rather than assuming that neurodiversity automatically strengthens such ecosystems, an open-innovation perspective raises a more exploratory question: how might differences in cognitive styles interact with boundary-spanning practices to shape the way knowledge and ideas are exchanged? Examining this interaction may help explain why similar neurodivergent characteristics can be experienced differently across roles and organizational settings.

To further interpret these dynamics, person-environment fit theory (Kristof-Brown et al., 2005) suggests that work experiences may depend partly on the compatibility between individual characteristics and environmental demands. Marketing environments that provide opportunities for creativity, flexibility, and varied forms of problem-solving may be experienced as more compatible with some ADHD-related characteristics (Verheul et al., 2015; Hatak et al., 2021). Broaden-and-build theory (Fredrickson, 2001) similarly emphasizes how positive social interactions may expand individuals’ resources for coping and engagement. Together, these perspectives suggest that the organizational consequences of neurodivergence should be examined in relation to contextual conditions rather than treated as inherent properties of ADHD. They therefore offer sensitizing perspectives for examining how role demands, social interaction, and organizational practices influence participants’ experiences of neurodiversity in marketing work.

Against this background, this study examines how marketing professionals experience and interpret the role of ADHD-related characteristics in team dynamics and creative work. More specifically, it asks: (1) How do marketing professionals experience and interpret the role of ADHD-related characteristics in team dynamics and creative work? (2) Under what organizational and customer-facing conditions do ADHD-related characteristics become perceived as enabling or constraining resources in marketing teams? Using an inductive grounded theory approach, the study develops an emergent conceptual framework based on participants’ experiences and interpretations. The aim is not to establish a causal effect of ADHD on innovation or performance, but to develop a theoretically informed account of the organizational and relational conditions through which ADHD-related characteristics may be experienced as either resources or constraints in marketing work.

## 2 Research background

### 2.1 Neurodiversity and its potential in workplaces

The neurodiversity paradigm, rooted in the social movements of the 1990s (Blume, 1998; Singer, 1999), has challenged traditional pathology-based views by redefining neurodevelopmental conditions such as attention deficit hyperactivity disorder (ADHD), autism, and dyslexia as part of the natural spectrum of human variation (Fletcher-Watson, 2022). Using a transdiagnostic approach, Apperly et al. (2024) found that divergent neurodevelopmental traits are described by a two-factor model comprising a general factor (N) and four condition-specific factors. This model emphasizes the presence of cognitive, behavioral, and social similarities across different neurodevelopmental conditions, providing a theoretical framework for analyzing the potential of these individuals in workplace settings.

Neurodiversity has been recognized as a potential source of innovation and creativity in organizations; Austin and Pisano (2017) argue that organizations that embrace neurodiversity have a competitive advantage, especially in industries that require unconventional thinking, such as marketing. This is due to neurodiverse individuals’ ability to offer unique perspectives and creative problem-solving (Krzeminska and Hawse, 2020). Relational challenges, including limitations in social skills and interpersonal interactions, can hinder full exploitation of this potential (Chapman, 2021). A study by van Rijswijk et al. (2024a, 2024b) found that these challenges are exacerbated in teams with high vertical differentiation (inequality in access to social resources), but that cross-functional activities can help reduce these barriers and enhance social skills. These findings are significant for marketing teams, where social interactions and interpersonal collaboration play a central role.

### 2.2 ADHD characteristics and their impact on creativity and innovation

Attention Deficit Hyperactivity Disorder (ADHD) related characteristics have been associated with creativity and innovation in some organizational and entrepreneurial contexts, particularly characteristics such as impulsivity, hyperactivity, and the ability to focus intensely on tasks of interest (Wiklund et al., 2016). Lerner et al. (2019), in a large-sample study (*N* = 9,869), demonstrated that individuals with ADHD were 1.7 times more likely to report entrepreneurial intentions and almost twice as likely to take entrepreneurial actions. These patterns have been discussed in relation to characteristics such as risk-taking and divergent thinking, which have been associated with ADHD in previous research (Nicolaou et al., 2011).

Research has demonstrated that impulsivity and hyperactivity, in contrast to inattention, are positively associated with entrepreneurial orientation and firm performance (Yu et al., 2021; Lerner et al., 2019; Verheul et al., 2015; Wiklund et al., 2017). Using person-environment fit theory and Broaden-and-build theory, Hatak et al. (2021) showed that entrepreneurs with ADHD symptoms perform better when they experience a passion for starting and developing a business (Antshel, 2018; Hatak et al., 2021). This passion attenuates the impact of ADHD weaknesses (e.g., inattention to detail) and amplifies its strengths (e.g., creativity and proactivity) (Yu et al., 2021). Moore et al. (2021) also showed that individuals with ADHD have a more intuitive cognitive style, consistent with entrepreneurial alertness and resource-based coping abilities, traits that are valuable in dynamic, customer-oriented marketing environments. Go Jefferies and Ahmed’s (2022) study suggests that neurodiversity, including ADHD, autism, and dyslexia, can be viewed not only as a potential challenge but also as a source of competitive advantage in the marketplace and workplace. Based on an analysis of Twitter data, the study identified five distinct segments of neurodiverse consumers, each with unique needs and characteristics. Some of these segments, such as scouted talents, have been targeted by leading companies for their specific skills (such as creativity, analytical thinking, and high focus). These findings can help marketing teams leverage these talents to innovate and improve customer engagement.

### 2.3 Customer-oriented boundary-spanning framework in marketing teams

Customer-oriented boundary-spanning behaviors (COBSB), introduced by Bettencourt and Brown (2003), focus on three key dimensions: external representation (creating a positive perception of the organization to customers), internal influence (sharing creative ideas with colleagues and managers), and service delivery (providing high-quality services). This framework provides a useful lens for examining innovation-related processes in marketing teams and emphasizes proactive interactions with stakeholders (Wagner et al., 2025). A study by Wagner et al. (2025) showed that customer-oriented boundary-spanning increases customer acceptance of innovative solutions by fostering information dissemination and focusing on the customer’s perspective. This is especially critical for marketing teams, whose success depends on the ability to respond quickly and creatively to customer needs.

Chien et al. (2021) found that frontline workers’ creativity, shaped by proactive personality, positively predicts boundary-spanning behaviors, whereas role stress can weaken these behaviors. Saeed et al. (2025) drew upon conservation of resources theory to contend that servant leadership and job meaningfulness can strengthen boundary-spanning behaviors, especially when employees’ work centrality is high. These findings highlight the importance of designing flexible roles and supportive work environments to harness the potential of neurodiverse employees, especially in marketing teams.

### 2.4 Theoretical contribution

Despite recent advances in the neurodiversity literature, few have specifically addressed the role of neurodiversity in marketing team dynamics (Go Jefferies and Ahmed, 2022). Most existing research has focused on human resource policies or workplace neurodiversity integration (Rao and Polepeddi, 2019), with less attention paid to the impact of neurodiversity on team innovation. In particular, the role of ADHD in facilitating creativity and performance in marketing teams—especially through a customer-oriented boundary-spanning framework—has been underexplored. Van Rijswijk et al. (2024b) found that boundary-spanning activities can help improve the social skills of neurodiversity. However, these mechanisms have not been empirically investigated in the marketing context.

Furthermore, although the association between ADHD and entrepreneurial orientation and creativity is well documented (Antshel, 2018; Lerner et al., 2019; Verheul et al., 2015; Wiklund et al., 2017), the causal mechanisms linking these traits to team performance in marketing remain unknown. This research gap is significant given the centrality of customer-oriented interactions and the need for marketing innovation. By focusing on the role of ADHD within marketing teams and drawing upon the customer-oriented boundary-spanning framework, the present study aims to fill these gaps and provide theoretical and practical insights for leveraging neurodiversity as a competitive advantage in dynamic, customer-oriented markets.

## 3 Research methodology

This study aims to investigate the role of neurodiversity, particularly attention-deficit/hyperactivity disorder (ADHD), in enhancing innovation in marketing teams through a customer-oriented boundary-spanning framework, using the classical grounded theory method (Glaser and Strauss, 1967). Due to its flexibility and ability to generate new theories from real data, this method is well-suited for exploring complex, less-studied phenomena, such as the impact of neurodiversity on team dynamics and innovation in marketing (Charmaz, 2014; Khan and Khan, 2024). The classical grounded theory approach was selected in this study because, first, the existing literature on the role of neurodiversity in marketing teams is limited and fragmented (Go Jefferies and Ahmed, 2022; van Rijswijk et al., 2024a), and there is a need to develop new theories based on empirical data. Second, the unique characteristics of neurodiversity (such as divergent thinking, risk-taking, and impulsivity) and the complexities of customer-oriented interactions in marketing require a method that enables free, in-depth exploration of the phenomenon (Urquhart, 2022).

Through simultaneous data collection and analysis, classical grounded theory enables researchers to identify hidden patterns and provides a theoretical framework for understanding the mechanisms that influence team innovation. This approach employs techniques including purposive sampling, theoretical sampling, semi-structured interviews, coding (open, selective, and theoretical), constant comparative analysis, and memoing (Chametzky, 2016; Chun Tie et al., 2019). Figure 1 visually represents the research process.

**Figure 1:**
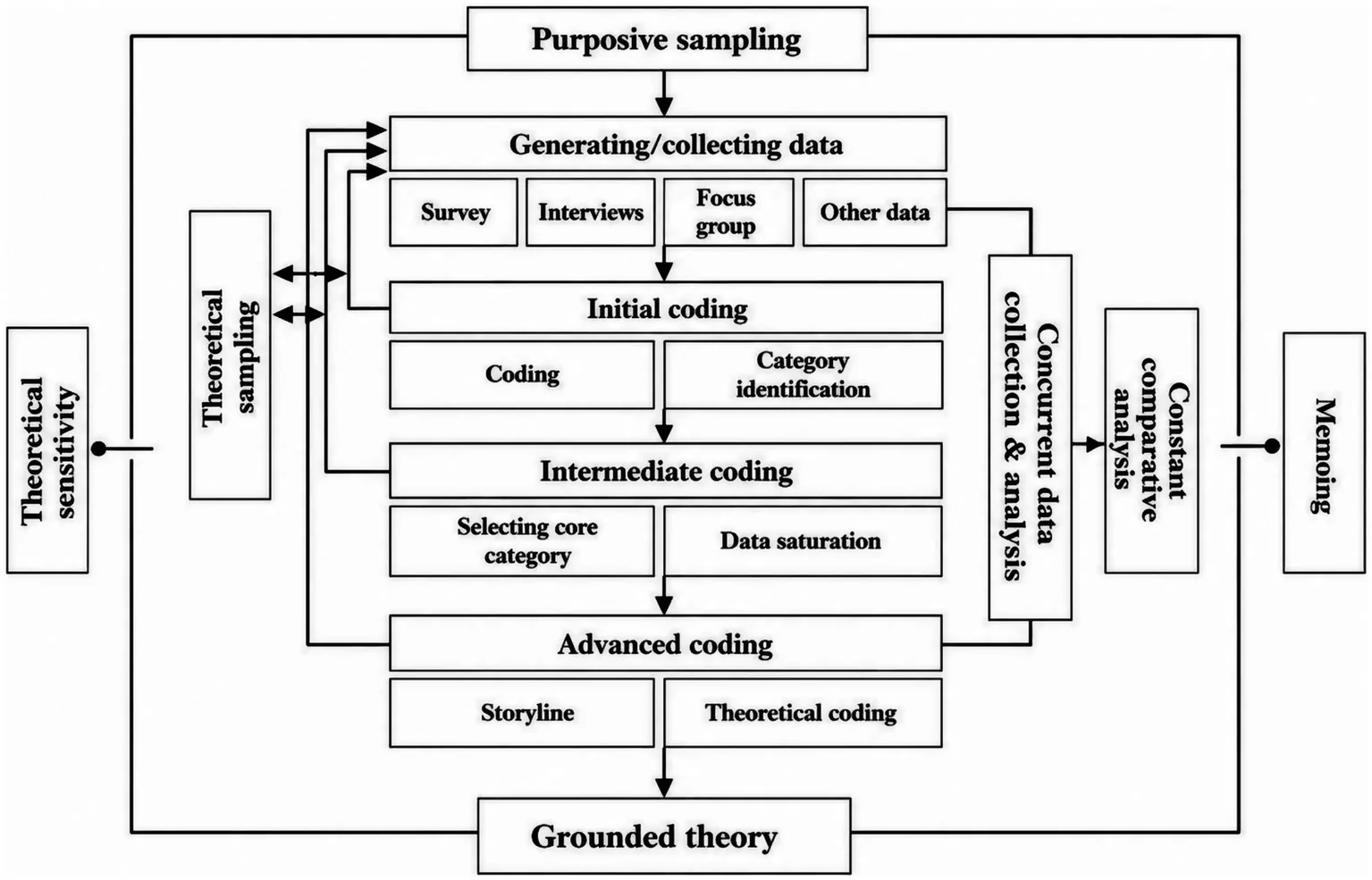
Research process (Chun Tie et al., 2019).

### 3.1 Sampling approach

This study employed a purposive and theoretical sampling approach to identify and select individuals who could provide rich insights into the role of neurodiversity, specifically attention-deficit hyperactivity disorder (ADHD), in fostering innovation in marketing teams. Purposive sampling was selected for its capacity to select participants with direct, relevant experience of the phenomenon under study. This method enabled the collection of rich, focused data, which is essential for exploring complex phenomena such as the impact of ADHD on team dynamics and on customer-focused boundary-spanning activities. Theoretical sampling was also conducted after the initial data collection phase and concurrently with the initial analysis. In this phase, participant selection was not based solely on predetermined demographic characteristics, but rather on concepts and categories that emerged as the data were continually compared. For example, when the role of organizational support, role fit, and customer-oriented interactions emerged in the initial data, the researchers sought to include participants who had direct and varied experience of these conditions. The purpose of this selection was not to increase sample diversity per se, but rather to expand the characteristics and dimensions of the emerging categories and examine their replicability or differences across conditions (Chun Tie et al., 2019).

### 3.2 Identifying and selecting participants with ADHD-related workplace experience

To ensure accurate identification of participants with ADHD and appropriate selection of participants with direct workplace experience of ADHD, professional networks and support organizations were contacted; several international and regional organizations were contacted, including ADHD associations in Europe (ADHD Europe), America (CHADD), and Asia (ADHD Singapore). A call for participation in the study was also published on professional platforms such as LinkedIn and ADHD-related Facebook groups, as well as through marketing. In these calls, it was explicitly stated that we were seeking individuals who:

1. Have received a formal diagnosis of ADHD from a medical professional or psychologist.
2. Have at least 3 years of professional experience in marketing teams (as a manager, front-line employee, or freelancer/consultant).
3. Are involved in customer-facing activities, such as developing advertising campaigns, managing customer interactions, or designing marketing strategies.

These criteria were designed to select individuals who could provide detailed, first-hand accounts of ADHD-related experiences in team dynamics and marketing innovation. To ensure the accuracy of the ADHD diagnosis, participants were asked to provide confirmation of their formal diagnosis (e.g., a medical certificate or psychological report) or to consent to contact a relevant professional. This process allowed us to ensure the validity of participants’ ADHD status and to avoid biases resulting from self-diagnosis.

### 3.3 Inclusion criteria and sample diversity

The study’s inclusion criteria were designed to include both individuals with ADHD and individuals with diverse organizational roles, industries, and geographic regions. Table 1 shows the inclusion criteria and their rationale:

**Table 1:** Participant inclusion criteria.

Entry criteria	Criteria details	Logic
Official ADHD diagnosis	Provide proof of an official ADHD diagnosis from a medical or psychological professional.	Ensuring data validity and focusing on people with real ADHD experience
Experience in marketing teams	At least 2 years of experience in marketing teams focused on customer-facing activities (manager, employee, freelancer, or consultant)	Ensure a deep understanding of team dynamics and customer-oriented interactions
Neurodiversity	Having a formal diagnosis of ADHD or another neurodevelopmental condition (by yourself or a team member)	Focus on experiences related to neurodiversity
Industrial diversity	Working in the advertising, digital marketing, or marketing consulting industries	Covering a wide range of creative marketing activities for the generalizability of findings
Geographic diversity	60% of the sample is from developed markets (Europe, America) and 40% from emerging markets (Asia, the Middle East)	Possibility of comparative comparison of the impact of ADHD in different cultural and economic contexts
Gender diversity	Balanced gender distribution (50% male, 50% female)	Reducing gender bias and increasing sample representation

In total, more than 50 responses to the initial calls were received. After careful consideration of the inclusion criteria and confirmation of the ADHD diagnosis, the final sample consisted of 35 participants, drawn from six countries: Iran (*n* = 8), the United States (*n* = 7), India (*n* = 6), Brazil (*n* = 5), the Netherlands (*n* = 5), and Japan (*n* = 4). Of these, 21 (60.0%) were from developed markets, and 14 (40.0%) were from emerging markets. The sample consisted of 15 marketing managers (42.9%), 12 marketing team members (34.3%), and 8 freelancers/consultants (22.9%), who were selected to provide sufficient diversity in work experiences and contexts and to allow for in-depth data analysis, in line with the recommendations of Eisenhardt and Graebner (2007). This geographic and occupational diversity was intended to broaden the contextual diversity of participants’ work experiences and to examine whether emerging theoretical concepts and relationships were also observed across different organizational and national contexts. The selection of countries was not done with the aim of achieving national representation or making systematic comparisons between countries; rather, the diversity of institutional and cultural contexts was used to strengthen theoretical sampling and explore and enrich emerging categories.

### 3.4 Participant profiles

Data were collected through 45- to 90-min semi-structured interviews, conducted online via Google Meet. Interviews were recorded and transcribed with informed consent from participants. The interview protocol was structured in three parts: (1) a demographic questionnaire to capture baseline information, (2) semi-structured questions about experiences related to neurodiversity, collaboration in marketing teams, customer-oriented interactions, and boundary-setting activities, and (3) follow-up questions to deepen and clarify experiences and perspectives raised during the interviews.

Given the international nature of the sample and potential differences in the equivalence and understanding of specialized concepts related to ADHD, neurodiversity, team dynamics, innovation, and customer-oriented interactions, all interviews were conducted in English. English was chosen as the common language of the study to avoid semantic differences resulting from translation and reinterpretation of specialized concepts in the data collection and analysis process. Therefore, none of the interviews were translated for the purposes of analysis, and data analysis was conducted directly based on the original English transcripts. In cases where participants’ responses contained ambiguous phrases or concepts, follow-up questions were used to clarify their intended meaning.

The demographic characteristics, professional role, ADHD status, and geographic distribution of participants are presented in Table 2. Participants included individuals with a formal diagnosis of ADHD and individuals without ADHD who had direct professional experience working with colleagues or team members with ADHD. This diversity allowed for constant comparison of narratives and examination of similarities, differences, and contextual conditions related to emerging themes.

**Table 2:** Overview of participants.

Professional group	Total, n (%)	Participants with ADHD, n (%)	Participants without ADHD, n (%)	Target industries	Geographical division, n (%)
Marketing managers	15 (42.9%)	8 (22.9%)	7 (20.0%)	Advertising: 5; Digital Marketing: 6; Consulting: 4	Developed markets: 9 (25.7%); Emerging markets: 6 (17.1%)
Marketing team members	12 (34.3%)	6 (17.1%)	6 (17.1%)	Advertising: 4; Digital Marketing: 5; Consulting: 3	Developed markets: 7 (20.0%); Emerging markets: 5 (14.3%)
Freelancers/consultants	8 (22.9%)	5 (14.3%)	3 (8.6%)	Advertising: 3; Digital Marketing: 3; Consulting: 2	Developed markets: 5 (14.3%); Emerging markets: 3 (8.6%)
Total	35 (100%)	19 (54.3%)	16 (45.7%)	—	Developed markets: 21 (60.0%); Emerging markets: 14 (40.0%)

The interview questions are presented in Table 3.

**Table 3:** Interview questions.

Number	Question
1	How has neurodevelopmental diversity (such as ADHD) impacted team dynamics in your marketing efforts?
2	What specific characteristics of ADHD have contributed to your team’s innovative processes?
3	How are customer-oriented cross-functional activities (such as interacting with customers or sharing ideas) implemented on your team?
4	What challenges have you experienced in social interactions or teamwork due to neurodiversity?
5	How does your organization support neurodiverse people to foster innovation?
6	Do you think ADHD has a positive or negative impact on customer-facing interactions?
7	How have digital tools or flexible work environments helped people with ADHD on your team?
8	What strategies have you employed to mitigate relational challenges related to neurodiversity in marketing teams?
9	How is customer feedback integrated into your team’s innovation processes?

### 3.5 Ethical considerations and sample quality

Informed consent was obtained from all participants before the interviews. They were assured that their identities and personal information would remain confidential and that the data would only be used for research purposes. For participants who reported a formal ADHD diagnosis, diagnostic verification was used to distinguish formally diagnosed participants from participants without ADHD and to reduce reliance on self-diagnosis. Also, to increase data validity, triangulation was used: supplementary information was collected from public sources (such as participants’ LinkedIn profiles or organizational websites) and, in 5 cases, interviews with colleagues or team members were conducted to provide additional perspectives on ADHD-related experiences, team interactions, and work practices. To ensure the quality of the sample and its appropriateness for scientific evaluations, the following measures were implemented:

1. Confirmation of ADHD diagnosis and participant ADHD status: The sample included both participants with a formal ADHD diagnosis and participants without ADHD. Among the 35 participants, 19 had a formal ADHD diagnosis, and 16 did not. Participants with a formal ADHD diagnosis were asked to provide official diagnostic documentation or to consent to confirmation according to the study’s eligibility procedure. Participants without ADHD were included when they had relevant professional experience working with, supervising, or collaborating with colleagues or team members with ADHD. The inclusion of participants without ADHD was intentional and was designed to capture workplace perspectives beyond first-person accounts. These participants had direct professional experience working with colleagues or team members with ADHD and were therefore able to provide observations concerning team interactions, work practices, collaboration, and organizational responses to ADHD-related characteristics. Participants with ADHD, in contrast, provided first-person accounts of their own experiences. These two perspectives were distinguished during data collection and subsequent analysis rather than being treated as analytically equivalent.
2. Diversity of participant perspectives: The sample was intentionally heterogeneous in terms of professional role, ADHD status, industry context, and geographic background. The inclusion of both participants with ADHD and participants without ADHD who had direct workplace experience with colleagues with ADHD enabled comparison across different experiential positions. Diversity in professional roles, industries, and geographic contexts was used to examine whether emerging categories remained relevant across different conditions rather than to establish statistical generalizability (Eisenhardt and Graebner, 2007).
3. Theoretical saturation: Theoretical saturation was not defined by a fixed, predetermined number of interviews, but rather was assessed using specific analytical criteria during simultaneous data collection and analysis, constant comparative analysis, and theoretical note-taking. Saturation was considered achieved when successive interviews: (1) did not generate new and meaningful conceptual codes; (2) did not add new features or dimensions to existing categories; (3) did not reveal new theoretical relationships between categories; and (4) did not fundamentally change the context, implications, or theoretical explanations of the core categories. These criteria were continually checked against new data and theoretical notes. With the six core categories established and the gradual decline of new theoretical information observed, the final six interviews were specifically examined as saturation confirmation interviews. Data from these interviews were compared with existing codes and categories to determine whether they generated new codes, features, dimensions, conditions, or theoretical relationships. These interviews largely resulted in repetition or enrichment of existing categories and did not generate new, substantive theoretical insights. Data collection was therefore stopped because the original categories had reached a sufficient level of theoretical development and the new data no longer provided meaningful changes to the theoretical structure or explanation. Consequently, the decision to saturate was made based on the reduction of new theoretical information and the consolidation of categories, rather than simply on achieving a predetermined sample size.
4. Transparency of the process: All steps in the selection of participants, including contacting support organizations and reviewing responses, were fully documented to ensure the replicability and transparency of the research.

### 3.6 Data analysis

Data collection and analysis were conducted simultaneously, following the logic of classical grounded theory (Glaser, 1998). The analysis process involved open, selective, and theoretical coding, with constant comparative analysis and theoretical note-taking throughout the research process. Rather than following a strictly linear sequence, the researchers iteratively moved between interview transcripts, initial codes, emerging categories, and theoretical insights. In the open coding phase, 304 codes were extracted from the interview transcripts. Then, through continuous comparison of codes in terms of conceptual similarities and differences, characteristics, conditions of emergence, and their relationship to the phenomenon under study, related codes were gradually grouped into 14 subcategories. Codes that referred to a common concept or process were grouped into a subcategory; subcategories that had meaningful conceptual overlap or theoretical connections were then compared with each other and merged into higher-order categories. In this process, integration was not based solely on the number or frequency of codes, but rather on the conceptual coherence, explanatory power, and relevance of the code set to the emerging theoretical process.

Next, 14 subcategories were compared and merged based on their conceptual features and theoretical relevance, resulting in the formation of six main categories. Selective coding focused the analysis on the categories that were most relevant to the emerging theoretical explanation; then, theoretical coding examined the relationships among the six main categories, resulting in the formation of three theoretical codes. Thus, the analytical path from the initial 304 codes to the 14 subcategories, six main categories, and three theoretical codes was iterative and grounded theory, and did not follow a predetermined classification system.

Constant comparative analysis and theoretical note-taking were used throughout these steps to refine the categories, identify their features and conditions, and guide subsequent data collection. Theoretical notes included emerging concepts, features, conditions, analytic comparisons, theoretical questions, and decisions to conduct further interviews. Narratives from participants with ADHD were also consistently compared with those from participants without ADHD who had direct professional experience working with colleagues or team members with ADHD. Similarities, differences, and conflicting narratives were used to refine the features and conditions of emerging categories.

Data coding and interpretation were conducted collaboratively by three researchers. Analytical disagreements were discussed and resolved through re-comparison with the original transcripts and analytical notes. Atlas.ti software was used to organize transcripts, codes, and analytic records; however, conceptual and theoretical decisions were made by the researchers.

### 3.7 Reflexivity of researchers

Given the interpretive nature of grounded theory research, the researchers recognized that their disciplinary backgrounds, prior assumptions, and interpretations could shape data collection, coding, constant comparison, and theoretical development. Reflexivity was therefore maintained throughout the research process through continuous memoing and discussion among the three researchers. During coding, emerging interpretations were repeatedly compared with the original interview transcripts and analytic memos to distinguish participants’ accounts from researchers’ interpretations. Differences in interpretation were discussed collaboratively and resolved through repeated examination of the underlying data rather than through reliance on a predetermined coding framework. The researchers also remained attentive to the possibility of interpreting ADHD-related characteristics primarily through a strengths-based perspective and therefore deliberately considered contradictory and challenging accounts, including impulsivity, distraction, stress, anxiety, communication difficulties, and social isolation. This reflexive process helped maintain alignment between the emerging theoretical framework and participants’ accounts.

## 4 Findings

### 4.1 Open coding

After the initial interview, the researcher began conducting interviews and open coding, transcribing and coding each interview as it was completed. Coding, as highlighted by Coyne and Cowley (2006), is a dynamic and reflexive process that involves assigning initial conceptual labels to the data (Coyne and Cowley, 2006; Glaser, 1998). Alongside data collection, the team conducted constant comparative analysis and memoing, which are integral components of grounded theory (Chun Tie et al., 2019). It is worth noting that these two activities were applied continuously throughout the research and were not limited to this specific phase. Through open coding, we identified 304 codes, which we then organized into 14 subcategories and six core categories. A sample of interviews and their initial codes is presented in Table 4.

**Table 4:** Illustrative verbatim interview excerpts and corresponding open codes.

Interview excerpt	Initial open code
*I have ADHD, and I think I take more risks than other people. This can be helpful because it allows me to embrace new opportunities. However, it is important to avoid making impulsive decisions and carefully evaluate the potential risks.*	Greater risk tolerance, avoiding impulsive decisions, and carefully assessing situations
*My experience has demonstrated me that people with ADHD need a strong support system to overcome the challenges that can hinder their functioning. A support network can provide emotional support, practical advice, and accountability, all of which are essential for success.*	Improving the support system, support network
*For teamwork, especially for people with ADHD, resilience is a vital trait. The ability to bounce back from setbacks and challenges is essential for long-term success. Whether we have a formal ADHD diagnosis or not, resilience must be ongoing.*	Resilience, the ability to bounce back from obstacles and challenges, continued resilience
*I complete my tasks more effectively when I receive continuous external validation, such as feedback or appreciation, than when I work independently. External validation helps compensate for deficits in executive function, such as self-motivation and focus, that are common in ADHD.*	External validation, feedback, or appreciation compensates for shortcomings in executive performance
*I feel that people with ADHD can generate ideas faster, although this may also involve a higher risk of errors.*	Faster idea generation alongside higher error rates
*The traditional hiring process was really hard for me. The vague interview questions prevented me from demonstrating my abilities effectively.*	Systemic Hiring Barriers for People with ADHD
*I believe people with ADHD often excel in emotional intelligence, empathy, and conflict resolution.*	Emotional intelligence, empathy, and conflict-resolution strengths
*If I’m in the wrong role, I will not be able to adapt to the work environment, and therefore, I will be more likely to seek a job where I have more control over my environment.*	Person-environment fit
*When faced with a risky activity, I tend to focus on its positive aspects and to be less sensitive to its potential negative aspects. Therefore, I am more likely to accept risky tasks.*	Risk-taking
*ADHD is often seen through its challenges: impulsivity, hyperactivity, and distractibility. But what if these same traits are actually strengths in the innovation-driven world? ADHD minds are designed to think outside the box, adapt to change, and solve problems in unexpected ways. Industries like technology, artificial intelligence, and cybersecurity need exactly that. Unfortunately, traditional education and work systems tend to ignore adaptability, the body’s preferences, and the untapped potential of people with neurological disorders. I think ADHD may not be a disorder at all, but rather a unique cognitive style for the future of work.*	Thinking outside the box, adapting to change, solving problems in unexpected ways, adaptability
*Team misunderstandings have sometimes led to my creative ideas being overlooked because colleagues perceive my behavior as unprofessional.*	Misunderstandings caused by unusual ADHD behaviors
*In busy meetings, I feel isolated because I cannot keep up with the pace of the discussions. This causes misunderstandings with colleagues.*	Social isolation in team meetings
*One of the most liberating things I learned from a coach is to separate what is in my control from what is not. I cannot control the market, the client’s budget, or any technical limitations. I can control how I communicate, the relationships I build, and whether I ask for help early.*	Separating controllable and uncontrollable issues, and how to communicate
*The severity of my ADHD symptoms makes it very challenging to manage stressful situations. My extreme impulsivity has led me to make several poor decisions in marketing campaigns.*	The intensity of impulsivity and its impact on marketing decision-making
*Being involved in multiple projects easily overloads my cognitive capacity, leading to stress and anxiety, which diminishes my performance. I need to organize and solve complexities to overcome stress and anxiety.*	Organizing and resolving complexities, stress, and anxiety under work pressure

Table 4 illustrates how participants’ own accounts were translated into initial open codes. The excerpts include both enabling and challenging experiences of ADHD in the workplace. Participants described creativity, divergent thinking, resilience, and adaptability, but also reported impulsive decision-making, distraction, stress and anxiety, communication difficulties, social isolation, and structural barriers. Thus, the coding process did not assume ADHD-related characteristics to be inherently positive or negative; rather, both dimensions emerged from participants’ accounts.

### 4.2 Selective coding

Glaser (1978) describes selective coding as a stage in which coding is limited to only those categories relevant to the research problem (Glaser, 1978). This process continues until both the main category and related categories reach saturation. Examples of open codes and their classifications, along with the results of selective coding, is presented in Table 5.

**Table 5:** Example of open codes and subcategories.

Main category	Subcategory	An example of an open source code
Cognitive dynamics and neurodiverse creativity	Divergent thinking	Creativity in brainstorming, divergent thinking in ADHD, intuitive cognitive style associated with ADHD, creativity related to neurodiversity, superior abilities, impulsivity intensity, and impact on marketing decision-making
	Risk-taking and creative impulsivity	Impulsivity and innovation, ADHD entrepreneurial tendency, ADHD enthusiasm for innovation, ADHD daydreaming
	Intense focus	Intense focus in ADHD, ADHD performance in dynamic environments, ADHD high work capacity, differences in intense focus
Synergistic connections	Team collaboration	Team dashboard, need for team transparency, increased collaboration, internal influence in teams, expansion of information in teams, challenges of team coordination across internal boundaries
	Communication challenges	Misunderstandings in communication, social challenges, misunderstandings in the team, double empathy problem, reducing relationship barriers, misunderstandings caused by unusual ADHD behaviors, and social isolation in team meetings
	Systemic barriers	Systemic hiring barriers for people with ADHD, structural discrimination in workplaces, organizational discrimination, and isolation
Customer-oriented neurodiverse dynamics	Role adaptation	Flexibility in roles, selection of aligned roles, adaptation to appropriate roles, and person-environment fit in ADHD
	Customer-oriented interactions	Customer relationship management, short interaction skills, and active listening
Resilient adaptability	Resilience to challenges	Reciprocal responsiveness, proactive interactions in boundary-spanning, customer-oriented segmentation for neurodiversity and ADHD resilience, ADHD resilience to failure, and ADHD energy adaptation
	Stress and anxiety management	ADHD-related anxiety, emotional regulation disorder, self-affirmation and mindset retraining, and ADHD lifestyle changes
Structured constructs	Organizational tools	External reminders, time blocking, task division, written instructions, visual aids, white space between meetings, and structuring days
	Distraction management	Difficulty concentrating, disorganization and forgetfulness, procrastination and time management, ADHD distraction, inattention, and ADHD performance challenges, and the impact of distraction severity on team productivity
External validation	Organizational support	Universal design, focusing on strengths, executive and public support, need for legal facilities, inclusive diversity space, de-stigmatization of ADHD, environmental facilities for ADHD, social support for ADHD, creating organizational support networks, feedback or appreciation
	Identity and self-confidence	Fear of disclosing diagnosis, disclosing diagnosis to management, ADHD strengths-based identity, ADHD support system

Selective coding consolidated the open codes into six main categories: Cognitive Dynamics and Neurodiverse Creativity, Synergistic Connections, Customer-Oriented Neurodiverse Dynamics, Resilient Adaptability, Structured Constructs, and External Validation. These categories captured both enabling and constraining dimensions of participants’ experiences. Creativity, divergent thinking, intense focus, adaptability, and resilience were described as potential resources, whereas communication difficulties, systemic barriers, distraction, stress and anxiety, and the need for organizational support reflected challenges that could constrain performance. The resulting categories therefore represent the conditions under which ADHD-related characteristics may support or hinder workplace functioning.

### 4.3 Theoretical coding

Theoretical coding integrated the six main categories into higher-order concepts and clarified their relationships within the emerging grounded theory (Glaser, 1978; Evans, 2013). This process resulted in three theoretical codes—Neurodiverse Cognitive Synergy, Mediated Empathy in Boundary-Spanning, and Resilient Organizational Adaptation—which capture the relationships among the main categories identified through selective coding. Specifically, Neurodiverse Cognitive Synergy integrates Cognitive Dynamics and Neurodiverse Creativity with Customer-Oriented Neurodiverse Dynamics; Mediated Empathy in Boundary-Spanning connects Synergistic Connections with External Validation; and Resilient Organizational Adaptation integrates Resilient Adaptability with Structured Constructs. These theoretical codes are presented in Table 6.

**Table 6:** Theoretical coding.

Theoretical code	Relationship between main categories
Neurodiverse cognitive synergy	This code refers to the dynamic process of integrating neurocognitive Cognitive synergy (focusing on ADHD cognitive traits such as divergent thinking and risk-taking) in marketing teams, leading to the generation of innovative ideas and customer-oriented solutions, and role adaptation. Customer-oriented neurodynamics, as a driving force for creativity, help mediate empathy across boundaries (customer-oriented and team interactions). This process is reinforced by external validation and helps create competitive value in customer interactions. Structured constructs facilitate these interactions.
Mediated empathy in boundary-spanning	This code describes mechanisms through which individuals with ADHD, through organizational support and identity and confidence building through structured tools (such as team dashboards and communication protocols) and addressing systemic barriers (such as reducing discrimination and systemic hiring barriers), reduce relational barriers (such as misunderstandings and social isolation), enhance team collaboration, and create synergistic bonds that facilitate customer -oriented interactions and role alignment. Empathy across boundaries depends on resilient organizational alignment, as individuals with ADHD need individual and team strategies and environmental supports to manage relational and organizational challenges and succeed in customer-oriented and team interactions.
Resilient organizational adaptation	The ability of individuals with ADHD to adapt to environmental and institutional challenges may depend on resilient flexibility and structured organizational supports. Such supports may help individuals manage work demands in dynamic marketing environments. This flexibility, particularly in the presence of symptom-related challenges such as distractibility and difficulties with emotional regulation, may facilitate the delivery of customer-oriented services within boundary-spanning activities when appropriate organizational conditions are present. Together with structured organizational supports, resilient adaptation may help individuals and teams manage these challenges and sustain effective functioning in dynamic marketing contexts. Resilient organizational adaptation may also contribute to the development of more inclusive work environments in which neurodiverse capabilities can be expressed and potentially support innovation. Under such enabling conditions, ADHD-related strengths may contribute to customer-oriented and boundary-spanning activities rather than being assumed to generate competitive advantage directly.

### 4.4 Grounded theory

The three theoretical codes formed an interconnected framework emerging from the interview data. Neurodiverse Cognitive Synergy captures how ADHD-related cognitive characteristics, including divergent thinking and intense focus, intersect with role adaptation and customer-oriented interactions. Mediated Empathy in Boundary-Spanning describes how communication and relational barriers between team members and across organizational and customer boundaries can be addressed through practices such as team transparency, communication protocols, feedback, and organizational support. Participants associated these practices with improved collaboration, reduced misunderstandings, stronger role alignment, and more effective customer-oriented interactions. Resilient Organizational Adaptation captures how individuals and teams respond to challenges such as distraction, stress, anxiety, and changing work demands through organizational tools, flexibility, and support. Together, these relationships support the model derived from the data (Figure 2) by presenting the following fundamental theory.

**Figure 2:**
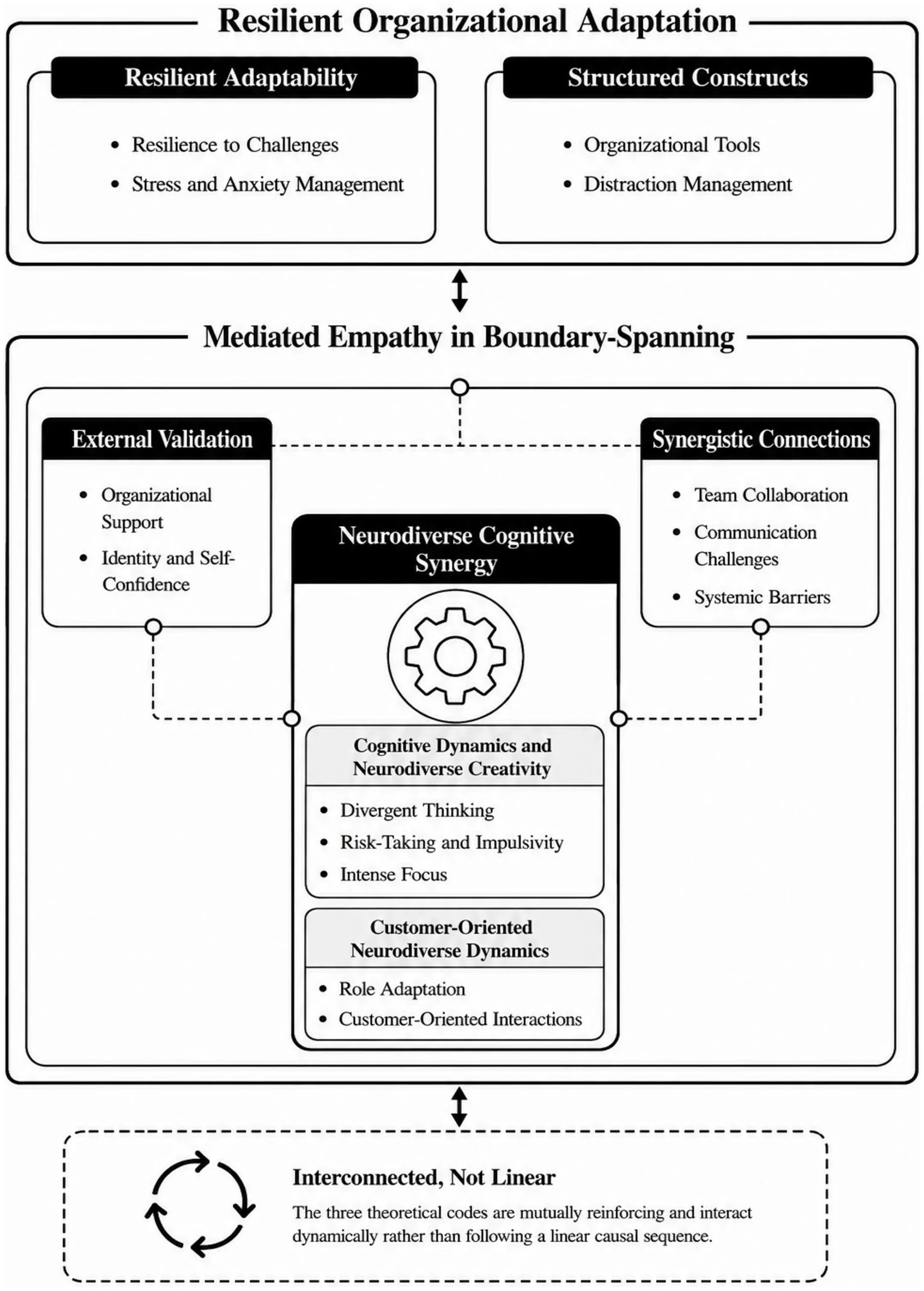
The role of neurodiversity in marketing teams through the Customer-oriented boundary-spanning framework.

Participants’ accounts suggest that “neurocognitive synergy and Mediated Empathy in Boundary-Spanning may be associated with resilient organizational adaptation in marketing teams and may support customer-oriented innovation under enabling organizational conditions.”

Figure 2 provides a visual representation of the grounded theory derived from the three theoretical codes of the study and depicts the relationships among them as interconnected and non-linear. At the center of the model, Neurodiverse Cognitive Synergy represents the cognitive synergy emerging from the interaction between ADHD-related characteristics, role demands, and customer-oriented contexts. This component comprises Cognitive Dynamics, Neurodiverse Creativity, and Customer-Oriented Neurodiverse Dynamics. The findings indicate that divergent thinking, risk-taking and impulsivity, and intense focus may function as potential resources for creativity and problem-solving under appropriate conditions, while their effectiveness remains contingent upon role fit and contextual conditions (Wiklund et al., 2016; Lerner et al., 2019; Hatak et al., 2021).

The surrounding layer, Mediated Empathy in Boundary-Spanning, represents the relational and interactional processes through which cognitive differences are translated into effective collaboration and interaction across team, organizational, and customer boundaries. Within this layer, External Validation comprises Organizational Support and Identity and Self-Confidence, highlighting the role of organizational support, feedback, and inclusive practices in strengthening participation and self-confidence. Synergistic Connections, in turn, comprises Team Collaboration, Communication Challenges, and Systemic Barriers, reflecting both opportunities for collaboration and the communication and organizational frictions identified in the data. Thus, mediated empathy refers to a relational process through which actors coordinate different perspectives and ways of working and transform these differences into workable forms of collaboration.

The upper layer, Resilient Organizational Adaptation, represents the organizational and individual mechanisms required to sustain and facilitate these processes and consists of Resilient Adaptability and Structured Constructs. Resilient Adaptability, represented by Resilience to Challenges and Stress and Anxiety Management, reflects the capacity to respond and adapt to workplace pressures and challenges. Structured Constructs, represented by Organizational Tools and Distraction Management, refers to the structures and tools that provide the stability and scaffolding required for effective functioning. This distinction suggests that realizing neurodiverse capabilities depends not only on individual adaptability but also on the alignment of organizational structures and support mechanisms with individual needs (Austin and Pisano, 2017; Hatak et al., 2021).

Overall, as indicated by Interconnected, Not Linear in the figure, the three theoretical codes should not be interpreted as sequential stages or linear causal relationships; rather, they dynamically interact with and mutually reinforce and shape one another. Accordingly, Neurodiverse Cognitive Synergy, Mediated Empathy in Boundary-Spanning, and Resilient Organizational Adaptation jointly shape the conditions under which ADHD-related cognitive capabilities may, depending on relational contexts, role demands, and organizational structures, be translated into creativity, adaptability, and customer-oriented interactions. The proposed model therefore avoids attributing innovation directly to individual ADHD characteristics and instead emphasizes the context-dependent and interactive process through which neurodiverse capabilities may be realized within marketing teams.

## 5 Discussion

### 5.1 Theoretical implications

This study contributes to the emerging literature on neurodiversity, marketing management, and organizational psychology by developing an exploratory, data-grounded framework connecting ADHD-related workplace experiences with customer-oriented work and innovation in marketing teams. The study addresses an underexplored intersection of ADHD, neurodiversity, marketing teams, customer-facing work, and organizational innovation. Drawing on qualitative accounts, the framework integrates three theoretical codes—Neurodiverse Cognitive Synergy, Mediated Empathy in Boundary-Spanning, and Resilient Organizational Adaptation—to explain how individual characteristics, interpersonal processes, and organizational conditions may interact in neurodiverse marketing environments. The contribution of the framework lies not in proposing that ADHD inherently produces innovation, but in showing how ADHD-related characteristics may become resources or challenges depending on task demands, role fit, interpersonal dynamics, and organizational support. This contextual interpretation extends the neurodiversity paradigm (Fletcher-Watson, 2022; Apperly et al., 2024) and complements person-environment fit theory (Kristof-Brown et al., 2005) by shifting attention from individual characteristics alone toward the organizational conditions under which those characteristics are experienced and utilized. It also responds to calls for greater attention to neurodiversity in organizational and marketing contexts (van Rijswijk et al., 2024a; Go Jefferies and Ahmed, 2022).

Neurodiverse Cognitive Synergy represents the first theoretical code. It captures participants’ accounts of divergent thinking, creativity, risk-taking, and intense focus in relation to creative and customer-oriented activities. This finding is consistent with research linking ADHD-related characteristics to entrepreneurship and creative activity (Wiklund et al., 2016; Lerner et al., 2019). Hatak et al. (2021) further demonstrate that the relationship between ADHD symptoms and entrepreneurial performance is contingent on other individual and contextual factors. The present study extends this contextual perspective from individual entrepreneurship to marketing-team processes by suggesting that cognitive differences may contribute to idea generation and customer-oriented problem solving when supported by appropriate roles and team arrangements. At the same time, the findings complicate a purely strengths-based interpretation. Participants also described impulsive decisions, distraction, stress, communication difficulties, and social isolation. For example, one participant reported that “my extreme impulsivity has led me to make several poor decisions in marketing campaigns,” while another stated, “In busy meetings, I feel isolated because I cannot keep up with the pace of the discussions.” These contrasting accounts suggest that the same ADHD-related characteristics may have divergent consequences across situations. Thus, the theoretical contribution of Neurodiverse Cognitive Synergy lies not in labeling ADHD characteristics as strengths, but in explaining their potentially contingent value within particular work contexts.

Mediated Empathy in Boundary-Spanning represents the relational dimension of the framework. It describes how communication and relational barriers associated with differences in work styles may be addressed through organizational support, structured communication, feedback, and interaction with internal and external stakeholders. Boundary-spanning activities are relevant because they connect team members with customers and other organizational actors and can facilitate the exchange of information and social resources (Bettencourt and Brown, 2003; Fredrickson, 2001; Wiklund et al., 2017). The concept also complements research on collective emotional intelligence (Curseu et al., 2015) by emphasizing that effective relational processes may depend not only on individual interpersonal capabilities but also on the structures through which interactions are organized.

Importantly, mediated empathy does not imply that individuals with ADHD inherently possess greater empathy or emotional intelligence. Rather, the concept refers to the processes through which empathy and mutual understanding may be facilitated when organizational practices reduce communication barriers and provide opportunities for clarification, feedback, and reciprocal adjustment. This interpretation is particularly relevant given participants’ reports of misunderstandings and social isolation alongside accounts of empathy and interpersonal engagement.

Resilient Organizational Adaptation represents the organizational dimension of the framework. Participants described the importance of role fit, autonomy, structured tools, feedback, and environmental support in managing ADHD-related challenges. This finding is consistent with person-environment fit theory (Kristof-Brown et al., 2005) and previous research emphasizing the importance of contextual conditions in understanding ADHD-related work and entrepreneurial outcomes (Verheul et al., 2015; Hatak et al., 2021). One participant explained, “If I am placed in the wrong role, I cannot adapt to the work environment, and therefore I am more likely to seek a job where I have more control over my environment.” This account illustrates that adaptation is not necessarily an individual responsibility; it may also involve organizational design, including flexibility, structured support, and appropriate allocation of responsibilities.

Taken together, the three theoretical codes suggest a multi-level process linking cognitive characteristics, relational processes, and organizational conditions. Neurodiverse Cognitive Synergy concerns the potential contribution of cognitive differences to creative and customer-oriented work; Mediated Empathy in Boundary-Spanning concerns the relational mechanisms through which differences can be negotiated; and Resilient Organizational Adaptation concerns the organizational structures that enable individuals and teams to manage these differences. The resulting framework therefore connects the individual, relational, and organizational levels rather than treating ADHD as an isolated individual characteristic.

This interpretation also contributes to marketing literature by connecting neurodiversity with organizational creativity (Amabile, 1996) and open innovation (Chesbrough, 2003). Neurodiverse Cognitive Synergy can complement creativity perspectives by highlighting how cognitive differences may contribute to idea generation, while Mediated Empathy in Boundary-Spanning connects neurodiverse team members with customers and other external stakeholders. These connections are theoretical extensions generated from the qualitative findings rather than empirical tests of the underlying creativity or open-innovation theories. The framework consequently offers a promising starting point for future research. Rather than claiming that ADHD causes innovation, it proposes that ADHD-related characteristics, relational processes, and organizational supports may form a configuration through which innovative potential can be realized in some marketing contexts. This proposition is consistent with prior research showing that ADHD-related characteristics can have both productive and challenging consequences depending on contextual conditions (Wiklund et al., 2016; Lerner et al., 2019; Hatak et al., 2021).

The findings also highlight several theoretical directions. First, cultural context may influence how ADHD-related characteristics are interpreted, disclosed, and supported. Because the present sample includes participants from multiple national contexts but is not designed for systematic cross-cultural comparison, the framework should not be assumed to operate uniformly across cultures. Second, the focus on ADHD means that the applicability of the framework to autism, dyslexia, dyspraxia, or other forms of neurodivergence remains an empirical question. Future research could examine whether the three theoretical codes are ADHD-specific or whether they can be adapted across neurodevelopmental conditions. Longitudinal and mixed-method research could further examine whether the relationships proposed by the framework are sustained over time and whether they are associated with observable outcomes such as innovation participation, team performance, retention, or inclusion.

### 5.2 Practical implications

This study also offers practical implications for marketing managers, HR professionals, and organizations seeking to develop more inclusive customer-oriented teams. Because the framework is exploratory, these implications should be viewed as context-sensitive organizational practices rather than empirically validated interventions.

First, organizations can combine flexibility with structure. Participants described potential benefits associated with creativity, divergent thinking, and intense focus, but also challenges involving distraction, impulsivity, stress, and workload management. Structured tools such as written instructions, task division, visual aids, time blocking, team dashboards, and appropriate digital systems may provide useful scaffolding (Chien et al., 2021).

Second, organizations can design roles around individual person-environment fit rather than assumptions about ADHD. Flexible responsibilities, opportunities for creative work, and greater control over task execution may benefit some employees, while others may prefer greater structure. The findings therefore support individualized job design rather than a standardized “ADHD role.”

Third, managers can strengthen feedback, communication, and relational support. Participants described both interpersonal strengths and communication difficulties. Regular feedback, transparent communication, written follow-up after meetings, and psychologically supportive team environments may help reduce misunderstandings and facilitate collaboration.

Fourth, organizations should adopt a balanced strengths-and-support approach. The findings do not suggest that organizations should simply “leverage ADHD strengths.” Instead, inclusion requires recognizing potential capabilities while also addressing challenges such as impulsivity, distraction, anxiety, stress, and social isolation. This balanced approach is particularly important because the same characteristic may function differently across roles and situations.

Finally, the findings suggest that neuroinclusion may be particularly relevant to customer-facing and boundary-spanning work, where diverse perspectives can interact with customer needs. However, organizations should not assume that employees with ADHD are naturally better suited to customer-facing or creative roles. Such assignments should be based on individual preferences, capabilities, and job requirements.

### 5.3 Limitations and future research

This study has several limitations that should be considered when interpreting the proposed framework. First, the sample size and purposive recruitment strategy are appropriate for grounded theory development but do not support statistical generalization. The recruitment process may also have introduced self-selection bias. Individuals who were particularly interested in neurodiversity, had salient workplace experiences of ADHD, or felt more comfortable discussing their experiences may have been more likely to participate. Consequently, the accounts may not fully represent individuals with less salient, less positive, or more difficult workplace experiences.

Second, the cultural composition of the sample may limit transferability across national contexts. Although participants were recruited from multiple countries, approximately 60% were from developed-market contexts. Organizational practices, attitudes toward hierarchy, disclosure, disability, and neurodiversity may vary across institutional and cultural environments. The findings should therefore not be interpreted as culturally universal. Future comparative research could examine whether the three theoretical codes operate differently across developed and emerging economies and across cultures with different expectations regarding hierarchy, autonomy, and disclosure.

Third, the study focuses specifically on ADHD and should not be generalized to neurodiversity as a whole. Autism, dyslexia, dyspraxia, and other neurodevelopmental conditions may involve different workplace experiences, support requirements, and patterns of strengths and challenges. Future research should examine whether the proposed framework is condition-specific or can be extended, with appropriate modification, to other neurodevelopmental conditions.

Fourth, ADHD may co-occur with other conditions that influence workplace experiences. The present study does not allow the independent contribution of ADHD to be separated from possible co-occurring conditions or individual differences. Future studies could explicitly examine how co-occurring conditions and other individual characteristics shape the relationships proposed in the framework.

Fifth, researcher subjectivity is an inherent consideration in grounded theory research. Although reflexive discussions, memoing, and collaborative comparison were used to reduce the influence of individual interpretations, researcher subjectivity cannot be eliminated in an interpretive grounded theory study. Future studies could strengthen the framework through independent coding, multiple researchers, triangulation with observations or organizational records, and mixed-method designs.

Sixth, the cross-sectional nature of the study limits conclusions about long-term organizational outcomes. The proposed framework identifies relationships among cognitive characteristics, relational processes, organizational support, and adaptation, but it does not establish whether these processes persist over time or whether supportive practices produce sustained changes in innovation, performance, retention, or inclusion. Longitudinal research could therefore examine how these relationships develop across different stages of employment and organizational change.

Seventh, confidentiality and stigma may have influenced participants’ accounts. Participants may have been reluctant to disclose experiences involving impairment, interpersonal conflict, failed decisions, anxiety, or negative workplace consequences when disclosure could carry social or professional risks. Conversely, participants who were more comfortable discussing neurodiversity may have emphasized particular aspects of their experiences. These possibilities should be considered when interpreting both positive and negative accounts.

Finally, the qualitative nature of the study means that the proposed relationships have not been empirically validated as causal relationships. The framework should therefore be understood as a theory-generating proposition that identifies potentially important relationships and conditions for further investigation. Future research could test these propositions using larger and more diverse samples, cross-cultural comparisons, longitudinal designs, and mixed methods.

## 6 Conclusion

Using a grounded theory approach, this study develops an exploratory, theory-generating frameworkneurodiversity, marketing managementfor understanding how ADHD-related workplace experiences may interact with marketing team processes and customer-oriented innovation. The findings identify three theoretical codes, Neurodiverse Cognitive Synergy, Mediated Empathy in Boundary-Spanning, and Resilient Organizational Adaptation, that capture patterned relationships across participants’ accounts.

The findings do not support a simple strengths-based or deficit-based interpretation of ADHD. Rather, participants described both potential resources, including divergent thinking, creativity, intense focus, adaptability, and risk-taking, and challenges involving impulsivity, distraction, stress, anxiety, communication difficulties, and social isolation. The findings therefore suggest that the consequences of ADHD-related characteristics are conditional and shaped partly by task demands, role fit, interpersonal relationships, and organizational support.

From a theoretical perspective, the study extends existing discussions of neurodiversity, person-environment fit, organizational creativity, and boundary-spanning by connecting individual characteristics with relational and organizational processes. Its principal contribution is not to demonstrate that ADHD causes innovation, but to propose a data-grounded framework for examining the conditions under which ADHD-related characteristics may be productively utilized in marketing teams. This interpretation is consistent with prior research showing that ADHD-related characteristics can have both enabling and constraining consequences and that their relationship with performance depends on contextual configurations (Wiklund et al., 2016; Lerner et al., 2019; Hatak et al., 2021).

Practically, the findings suggest that organizations may consider combining autonomy and flexibility with structured support, transparent communication, supportive feedback, and appropriate role alignment. These implications should be understood as context-sensitive organizational design considerations rather than validated interventions. Inclusive management should recognize variation among employees with ADHD rather than assuming that all individuals share the same strengths or support needs.

The study is subject to limitations concerning sample size, self-selection bias, cultural composition, the exclusive focus on ADHD, possible co-occurring conditions, researcher subjectivity, cross-sectional design, confidentiality, and stigma. These limitations constrain the generalizability and causal interpretation of the framework. Future research should therefore test and refine the proposed relationships using larger, more culturally diverse samples; longitudinal and mixed-methods designs; and comparisons across neurodevelopmental conditions and organizational contexts.

Ultimately, this study invites researchers and organizations to move beyond both purely deficit-based and overly celebratory accounts of ADHD. Rather than treating neurodiversity as inherently beneficial or problematic, the findings suggest examining how organizational conditions shape how neurodivergent characteristics are experienced and utilized. In this sense, the framework offers a starting point for further empirical investigation into neuroinclusive marketing teams and the organizational conditions that may support innovation.

## Data Availability

The raw interview data are not publicly available because they contain confidential and potentially identifiable information and were collected under ethical and confidentiality commitments. De-identified interview excerpts supporting the findings are included in the manuscript. The interview protocol, methodological procedures, and other non-identifiable materials are described in the manuscript and supplementary materials and may be made available upon reasonable request to the Faculty of Economics, Management and Administrative Sciences, Semnan University, subject to appropriate ethical and confidentiality considerations.
